# Mixed pathologies and cognitive outcomes in older persons ‘possibly’ eligible for anti‐amyloid treatment: a community‐based study

**DOI:** 10.1002/alz.086333

**Published:** 2025-01-03

**Authors:** Alifiya Kapasi, Bryan D James, Lei Yu, David A. Bennett, Patricia A Boyle, Julie A. Schneider

**Affiliations:** ^1^ Rush Alzheimer’s Disease Center, Chicago, IL USA; ^2^ Rush Alzheimer’s Disease Center, Rush University Medical Center, Chicago, IL USA

## Abstract

**Background:**

The recent approval of two anti‐amyloid antibodies, Aducanamab and Lecanamab, have set the stage for the next generation of anti‐amyloid treatments. Despite the capability of these treatments to lower Aβ brain levels, there is thus far limited clinical efficacy on cognitive outcomes. Because eligibility for treatment includes individuals with MCI or mild dementia, that often harbor mixed pathologies, the cognitive impact of other brain pathologies may be important. This study investigated mixed brain pathologies from autopsied persons that would be considered eligible for anti‐amyloid treatment and the association of pathologies with rate of decline in global cognition and five cognitive domains.

**Method:**

Eligibility was defined based on having an MMSE score ≥ 20, a clinical diagnosis of MCI or Alzheimer’s dementia, and a level of Aβ pathology that would be indicative of having a positive amyloid PET scan (CERAD score≥moderate). We examined the number and types of co‐pathologies. Mixed‐effect models were employed to examine association of pathologies (β‐amyloid, tangles, LATE‐NC, infarcts, and LB) with rate of decline in global cognition and across 5 cognitive domains.

**Result:**

Among 428 older autopsied persons (mean age at death = 90 years) considered possibly eligible for anti‐amyloid treatment, 58% had MCI and 42% had mild dementia. The majority (94%) had a pathologic diagnosis for AD, of which most had ≥ 1 co‐pathology; 43% had +1 co‐pathology, 22% had +2 co‐pathologies, and 3% had +3 co‐pathologies. Regarding type of co‐pathologies, 37% had AD + infarcts, 36% had AD + LATE, and 24% had AD + LB. In mixed‐effect models, tangles were associated with a faster rate of global cognitive decline, specifically in the domains of episodic (estimate = ‐0.04; SE = 0.01; p<0.001) and semantic memory (estimate = ‐0.02; SE = 0.01; p = 0.001). Independent of tangles, LATE‐NC and β‐amyloid were also associated with decline in episodic (estimate = ‐0.03, SE = 0.01; p = 0.004) and semantic memory (estimate = ‐0.02; SE = 0.01, p = 0.04), respectively. Infarcts and LB were not associated with decline in global cognition or any cognitive domains.

**Conclusion:**

Mixed pathologies are common in this group of community‐dwelling older persons possibly eligible for anti‐amyloid treatments. Tangles and LATE‐NC pathologies drive late‐life cognitive decline, especially episodic memory.

